# Dependence, plans to quit, quitting self-efficacy and past cessation behaviours among menthol and other flavoured cigarette users in Europe: The EUREST-PLUS ITC Europe Surveys

**DOI:** 10.18332/tid/111356

**Published:** 2019-09-19

**Authors:** Aleksandra Herbeć, Mateusz Zatoński, Witold A. Zatoński, Kinga Janik-Koncewicz, Ute Mons, Geoffrey T. Fong, Anne C. K. Quah, Pete Driezen, Tibor Demjén, Yannis Tountas, Antigona C. Trofor, Esteve Fernández, Ann McNeill, Marc Willemsen, Constantine I. Vardavas, Krzysztof Przewoźniak, on behalf of the EUREST-PLUS consortium

**Affiliations:** 1 Health Promotion Foundation (HPF), Warsaw, Poland; 2 Department of Behavioural Science and Health, University College London, London, United Kingdom; 3 Centre for Behaviour Change, Clinical, Educational and Health Psychology, University College London, United Kingdom; 4 European Observatory of Health Inequalities, President Stanisław Wojciechowski State University of Applied Sciences, Kalisz, Poland; 5 Tobacco Control Research Group, Department for Health, University of Bath, Bath, United Kingdom; 6 Cancer Prevention Unit and WHO Collaborating Centre for Tobacco Control, German Cancer Research Center (DKFZ), Heidelberg, Germany; 7 Department of Psychology, University of Waterloo (UW), Waterloo, Canada; 8 School of Public Health and Health Systems, University of Waterloo (UW), Waterloo, Canada; 9 Ontario Institute for Cancer Research, Toronto, Canada; 10 Smoking or Health Hungarian Foundation (SHHF), Budapest, Hungary; 11 National and Kapodistrian University of Athens (UoA), Athens, Greece; 12 University of Medicine and Pharmacy ‘Grigore T. Popa’ Iasi, Iasi, Romania; 13 Aer Pur Romania, Bucharest, Romania; 14 Tobacco Control Unit, Catalan Institute of Oncology (ICO), and Tobacco Control Research Group, Bellvitge Biomedical Research Institute (IDIBELL), Catalonia, Spain; 15 School of Medicine and Health Sciences, University of Barcelona, Catalonia, Spain; 16 UK Centre for Tobacco and Alcohol Studies, London, United Kingdom; 17 National Addiction Centre, King’s College London, London, United Kingdom; 18 Department of Health Promotion, Maastricht University, Maastricht, the Netherlands; 19 European Network for Smoking and Tobacco Prevention (ENSP), Brussels, Belgium; 20 University of Crete (UoC), Heraklion, Greece; 21 Maria Skłodowska-Curie Institute - Oncology Center, Warsaw, Poland

**Keywords:** menthol cigarettes, Europe, smoking cessation, attitudes, cross-sectional

## Abstract

**INTRODUCTION:**

This study characterises smoking and cessation-related behaviours among menthol and other flavoured cigarette users in Europe prior to the implementation of the European Tobacco Products Directive (TPD) ban on the sale of flavoured cigarettes.

**METHODS:**

An analysis of cross-sectional data from the 2016 EUREST-PLUS ITC Europe Surveys was conducted among a sample of 10760 adult smokers from eight European Union Member States. Respondents were classified as menthol, other flavoured, unflavoured, or no usual flavour cigarette users and compared on smoking and cessation behaviours and characteristics. Data were analysed in SPSS Complex Samples Package using bivariate and multivariate regression analyses adjusted for sociodemographic characteristics, dependence, and country.

**RESULTS:**

In bivariate analyses, cigarette flavour was significantly associated with all outcomes (p<0.001). After adjusting for sociodemographic characteristics, these associations attenuated but remained significant and in the same direction for dependence, self-efficacy, plans to quit, past quit attempts, and ever e-cigarette use. In fully adjusted models, compared to smokers of non-flavoured cigarettes, menthol smokers were less likely to smoke daily (AOR=0.47, 95% CI: 0.32–0.71), smoke within 30 min of waking (0.52,0.43–0.64), consider themselves addicted (0.74,0.59–0.94), and more likely to have ever used e-cigarettes (1.26,1.00–1.57); other flavoured cigarette smokers were less likely to smoke daily (0.33,0.15–0.77), and have higher self-efficacy (1.82,1.20–2.77); no usual flavour smokers were less likely to smoke daily (0.34,0.22–0.51), smoke within 30 min of waking (0.66,0.55–0.80), consider themselves addicted (0.65,0.52–0.78), have ever made a quit attempt (0.69,0.58–0.84), have ever used e-cigarettes (0.66,0.54–0.82), and had higher self-efficacy (1.46,1.19–1.80).

**CONCLUSIONS:**

Smokers of different cigarette flavours in Europe differ on smoking and cessation characteristics. The lower dependence of menthol cigarette smokers could lead to greater success rates if quit attempts are made, however cross-country differences in smoking behaviours and quitting intentions could lead to the TPD ban on cigarette flavours having differential impact if not accompanied by additional measures, such as smoking cessation support.

## INTRODUCTION

The 2014 European Tobacco Product Directive (TPD)^1^ introduced a ban on the sales of menthol and flavoured cigarettes (MFCs) in the European Union member states (EU MS), with a transitional period for menthol cigarettes until May 2020^2^. This ban aims to limit appeal and attractiveness of such cigarettes, particularly to young people, and could constitute an important opportunity to promote cessation among MFC smokers^3^. To date, most studies of menthol cigarette smokers have been conducted in the United States^4-10^, with little research available on European smokers^11^. The International Tobacco Control (ITC) Project conducted in eight European countries offers an opportunity to study the characteristics and behaviours of MFC smokers^12^. An earlier study using this sample has shown important differences in the prevalence of flavoured cigarette use between different European countries, as well as considerable differences in attitudes towards tobacco control policies between users of different cigarette flavours^13^. The present study extends this analysis to characterise the smoking and past cessation behaviours, motivation and self-efficacy to quit among MFC smokers in Europe.

A considerable minority of smokers use flavoured cigarettes, with menthol being the most popular^13-15^. The prevalence of menthol cigarette smoking in the United States among past 30-day smokers in 2012–2014 was 39%^5^, with the rates being highest among African-American smokers^16^. Prevalence of MFC smokers in Europe varies considerably across countries, and ranges from 6% in Spain to 15% in the UK^13^. MFCs are also more commonly used among younger smokers, less-established or novice smokers, and those who are experimenting with smoking^14,17^. MFCs have also been considered a gateway product, especially as some research suggests that switching from menthol to non-menthol cigarettes is more common than switching from non-menthol to menthol cigarettes^18^. Furthermore, prior research conducted in the US has pointed to higher levels of cigarette dependence among menthol cigarette smokers than unflavoured cigarette smokers^19-21^, although some studies have found no difference^22^.

The popularity of MFCs has been attributed to several factors. Flavouring, and especially menthol that has cooling and anaesthetic effects, can improve the smoking experience by masking or limiting some of the negative sensations associated with smoking, such as burning, throat pain, and cough^14^. The tobacco industry has been manipulating the menthol content of cigarettes to promote smoking initiation and sustain nicotine dependence^23,24^, and has actively advertised menthol brands, especially among ethnic groups and younger smokers in the US^25^. Analysis of tobacco industry research has also suggested that while younger and less experienced smokers may prefer menthol cigarettes due to the less harsh smoking experience, more dependent and experienced smokers may seek the menthol flavours for their strong sensory qualities^26^. Finally, menthol in cigarettes may improve nicotine intake, thus helping to maintain high enough nicotine levels among smokers who cannot afford to purchase and smoke more cigarettes per day^19^.

The aim of the present study was to cross-sectionally assess MFC smokers in eight of the EU MS on cigarette dependence, plans to quit and quitting self-efficacy, and past cessation behaviours prior to the implementation of the TPD. The findings could help inform tobacco control policies and smoking cessation campaigns to accompany the TPD ban on the sale of flavoured and menthol cigarettes.

## METHODS

### Design

This study was conducted within the context of the European Commission Horizon 2020 funded study entitled European Regulatory Science on Tobacco: Policy implementation to reduce lung diseases (EUREST-PLUS-HCO-06-2015). Cross-sectional analysis was conducted of data from current smokers aged ≥18 years from eight European EU MS participating in the ITC Project^27^. The countries included in the study were: Germany, Greece, Hungary, Poland, Romania and Spain (part of the EUREST–PLUS ITC Project, later referred to as 6E^28-30^), the Netherlands^31,32^, and England^30,33,34^. The survey in 6E was conducted using computer-assisted personal interviews (CAPI) and in the Netherlands and England through a web survey.

### Sampled populations

The total sample analysed in this study comprised 10760 adult smokers from the eight European countries (n=6011 from the six countries of the ITC 6E Survey, n=3536 from ITC England, and n=1213 from ITC Netherlands, see details below). This sample was aged 43.2 (SE=0.19) years on average, 43.5% female, and 14.0% had higher education (for a detailed description of the sample, including the breakdown of prevalence of smoking of different cigarette flavours across sociodemographic groups and countries, see Zatoński et al.^13^ in the same supplement).

The ITC 6E Survey was conducted between 18 June 2016 and 12 September 2016^12,28^. The surveyed sample comprised a nationally representative sample of adult cigarette smokers aged ≥18 years (about 1000 participants in each country). Sampling was done using geographical strata determined by Nomenclature of Territorial Units for Statistics (NUTS) regions crossed with degree of urbanization (urban, intermediate, rural). Approximately 100 area clusters were sampled in each country (allocated to strata proportionally to population size for people aged ≥18 years), with the aim of recruiting 10 adult smokers per cluster. Within each cluster, household addresses were sampled using a random walk design. One randomly selected male smoker and one randomly selected female smoker were chosen for interview from a sampled household, where possible. Screening of households continued until the required number of smokers from the cluster had been interviewed.

Data from England was nationally representative and came from Wave 1 of the ITC Four Country Smoking and Vaping Survey conducted between July and November 2016^30,33,34^. Further details on the survey methodology are available elsewhere^13,35^. Only current adult smokers aged ≥18 years were included in the present analysis (n=3536).

Data for Wave 10 of the ITC Netherlands (NL) Survey^31,32^ were collected between November and December 2016. Respondents were 1696 adults aged ≥15 years recruited as cigarette smokers, who were part of a probability-based web database^36^. The nationally representative sample included 1318 respondents who had also participated in Wave 9 and 378 new respondents recruited to replenish dropouts^37^. Only current adult (aged ≥18 years) smokers were included in the analysis (n=1213).

### Measurements

#### Cigarette flavour

Participants were asked if they had a usual cigarette brand and if they did, they were asked about the flavour of their usual brand: just tobacco (‘unflavoured tobacco’), tobacco and menthol (‘menthol’), or ‘other flavoured’. A final category of ‘no usual flavour’ included all remaining participants who either: a) reported having no usual brand, b) refused to answer or answered ‘don’t know’ to the question about having a usual brand, or c) reported having a usual brand but who did not provide information on the flavour of that brand.

#### Smoking dependence

Dependence was assessed using: i) smoking daily vs non-daily, ii) cigarettes smoked per day, iii) time to first cigarette, and iv) Heaviness of Smoking Index (HIS), scores ranging 0–6, with ≥4 suggesting greater dependence^38^; as well as self-perceived addiction levels (‘How addicted are you to cigarettes?’ with response ‘not at all’, ‘yes-somewhat addicted’, or ‘yes-very addicted’).

#### Motivation to quit

Respondents were asked:

‘Are you planning to quit smoking? within the next month/within the next 6 months/beyond 6 months/not at all/don’t know’. In the logistic regression the responses were dichotomised to ‘planning to quit in the next 6 months’ vs ‘all other’^39-41^.

#### Quitting self-efficacy

This involved two questions^42^ that assessed:

1. confidence to quit (‘If you decided to give up smoking completely in the next 6 months, how sure are you that you would succeed? Not at all sure or slightly sure/moderately sure/very sure or extremely sure’)^43,44^. In the logistic regression, the answers were dichotomised to ‘very or extremely sure’ vs ‘all other’; and
2. perceived difficulty of quitting (‘How difficult would it be for you to quit smoking if you wanted to? not at all/slightly/moderately/very/extremely’)^45,46^. For the purpose of logistic regression the answers were dichotomised to ‘not at all or slightly vs all other’.

#### Prior quit attempts and use of cessation aids

Data were collected on: i) having ever made a quit attempt (yes/no), and ii) having made a quit attempt in the past 12 months (yes/no). Participants who reported having made a quit attempt were asked about the use of any cessation aids during the last quit attempt (yes/no; combining any of nicotine products, medication on prescription, face-to-face support, quitline, cessation services, online support, and e-cigarettes). Due to only very few cases of refused to answer/don’t know or missing data for these variables, the data are reported as ‘yes’ vs ‘all other’. All participants were also asked about ‘ever hearing about e-cigarettes’, and those who responded affirmatively (n=8870) were asked about having ever used e-cigarettes; for the present analysis the variable on ever e-cigarette use was dichotomised into yes/no (with no including all other answers and participants who had not heard about e-cigarettes).

#### Sociodemographics (confounders and covariates)

The following sociodemographic variables were assessed: country, sex (male/female), age group (18–24/25–39/40–54/≥55 years), educational attainment (derived and standardised variable across countries: low/medium/high)^47^.

### Statistical analysis

For the main analysis, data from all countries were pooled and analysed together using the Complex Samples package in SPSS 23.00 that accounted for the sampling procedure in each country. Sampling weights were also used to ensure results represent the population of smokers in each country. Missing data were not imputed (for sociodemographic and HSI variables, the missing data ranged from 0% for age and 7.2% for HSI; for independent variables missing data ranged from 0% for daily smoking to 17.5% for ever e-cigarette use). ‘Refused to answer/don’t know’ responses to individual questions were included in the ‘no’ category for dichotomous variables. Bivariate analyses (crosstabs for categorical and general linear models for continuous variables) compared smokers of different cigarette flavours on sociodemographic, smoking, quitting, and attitudinal variables. We present percentages and means and 95% confidence intervals (CI). Unless indicated, weighted number of participants are provided, rounded up to the nearest whole number.

We conducted unadjusted and adjusted logistic regression models to assess the relationship between cigarette flavour and categorical outcomes of interest. In all analyses, smokers of unflavoured tobacco were the reference group. The dependent variables were dichotomised outcomes of interest (being a daily smoker vs non-daily; smoking within the first 30 min of waking vs all other; HSI of ≥4 vs lower; considering oneself to be very addicted vs all others; ever having made a quit attempt vs not; having made a quit attempt in the past 12 months vs not; having used quit aids and e-cigarettes in the last quit attempt vs not; planning to quit in the next 6 months vs not; being very or extremely sure one could quit vs not; expecting quitting to be very or extremely difficult vs not. Findings from three models are reported: unadjusted (Model 1), partially adjusted for sociodemographic characteristics and dependence level (categorical variables: sex, age group, education level, and continuous variable HSI; Model 2), and fully adjusted with country variable added as an additional adjustment (Model 3). We report associated odds ratios (ORs) and 95% confidence intervals (95% CIs). The multivariable regression models assessed main effects, with all variables entered together. To adjust for multiple comparisons and familywise errors (about 10 tests for related outcomes), we used the Sidak correction to adjust alpha to <0.005 as a threshold for statistical significance.

## RESULTS

The majority of smokers (88.1%) had a preferred cigarette brand. Unflavored tobacco was smoked by 77.4%, menthol by 7.4%, other flavoured by 2.9%, and 12.3% had no usual flavour. Table 1 presents results of bivariate comparisons of smokers of different cigarette brand flavours on cigarette dependence, as well as on smoking and quitting behaviours and attitudes.

**Table 1:** Smoking and cessation behaviour among smokers of different cigarette flavours in ITC European countries

*Behaviour*	*Menthol*	*Other flavoured*	*Unflavoured tobacco*	*No usual flavour*	*Total*	*p*
**Cigarette dependence**						
**Smoking daily % (95% CI)**	78.8 (74.8–82.3)	89.3 (83.3–93.3)	94.0 (93.3–94.6)	79.8 (76.7–82.6)	90.9 (90.2–91.7)	**<0.001**
**Cigarette smoked/day, % (95% CI)** ^**a**^						
≤10	64.5 (60.4–68.4)	36.7 (29.6–44.5)	37.3 (36.0–38.8)	47.0 (43.1–51.2)	40.5 (39.3–41.8)	**<0.001**
11–20	30.7 (27.0–34.6)	49.6 (41.6–57.6)	50.0 (48.6–51.3)	39.1 (35.3–43.0)	47.2 (46.0–48.5)	
21–30	2.9 (1.9–4.5)	7.2 (4.6–11.2)	9.1 (8.3–9.9)	9.4 (7.4–11.8)	8.6 (7.9–9.3)	
≥31	1.8 (1.0–3.3)	6.4 (3.4–11.9)	3.6 (3.1–4.2)	4.4 (3.2–6.1)	3.7 (3.2–4.2)	
**Minutes to first cigarette, % (95% CI)^b^**						
>60	36.2 (31.7–40.9)	19.3 (13.1–27.3)	18.0 (16.9–19.1)	25.2 (21.7–29.0)	20.1 (19.0–21.2)	**<0.001**
31–60	16.7 (13.6–20.4)	17.7 (13.0–23.7)	16.2 (15.2–17.3)	16.8 (14.0–20.0)	16.4 (15.4–17.4)	
6–30	33.9 (29.6–38.4)	46.7 (39.4–54.1)	45.1 (43.6–46.7)	36.7 (32.7–40.1)	43.5 (42.0–44.8)	
≤5	13.2 (10.3–16.8)	16.3 (10.0–25.6)	20.7 (19.4–22.1)	21.7 (18.5–25.3)	20.2 (19.0–21.4)	
**HSI, Mean (95% CI)^c^**	1.70 (1.56–1.85)	2.49 (2.22–2.75)	2.50 (2.46–2.55)	2.36 (2.21–2.50)	2.43 (2.39–2.47)	**<0.001**
**Consider themselves very addicted, % (95% CI)**	28.7 (25.0–32.7)	34.6 (26.9–43.2)	44.0 (42.5–45.6)	30.5 (27.4–33.8)	41.0 (39.6–42.3)	**<0.001**
**Plans and attitudes on quitting**			**% (95% CI)**			
**Plans to quit in the next 6 months^d^**	31.1 (27.1–35.5)	17.5 (12.2–24.5)	20.3 (19.2–21.4)	20.4 (17.7–23.4)	21.0 (20.0–22.0)	**<0.001**
**Plans to quit in the future**						
In the next month	12.9 (10.0–16.4)	3.1 (1.5–6.1)	6.5 (5.8–7.2)	7.7 (5.9–10.0)	7.0 (6.4–7.7)	**<0.001**
Next 6 months	18.3 (15.1–22.0)	14.4 (9.7–21.0)	13.8 (12.9–14.8)	12.7 (10.6–15.0)	14.0 (13.2–14.9)	
Beyond 6 months	33.7 (29.8–37.9)	33.3 (25.8–41.8)	31.4 (30.0–32.8)	30.5 (27.4–33.9)	31.5 (30.3–32.8)	
Not planning to quit	27.0 (23.3–31.1)	42.7 (32.9–53.1)	40.6 (39.1–42.1)	39.6 (35.8–43.7)	39.6 (38.2–40.9)	
Don’t know	8.1 (6.1–10.6)	6.5 (3.1–13.0)	7.7 (7.0–8.5)	9.5 (7.3–12.3)	7.9 (7.2–8.7)	
**SEF: confidence to quit (sure would succeed at quitting)^e^**						
Not at all or slightly sure	50.3 (45.8–54.8)	62.5 (55.1–69.4)	63.8 (62.4–65.1)	52.6 (48.5–56.2)	61.4 (60.1–62.2)	**<0.001**
Moderately sure	30.6 (26.6–35.0)	21.6 (16.5–27.8)	23.2 (22.0–24.5)	27.3 (23.9–31.0)	24.2 (23.1–25.4)	
Very or extremely sure	19.1 (15.8–22.9)	15.9 (10.8–22.9)	13.0 (12.0–14.0)	20.3 (17.3–23.8)	14.4 (13.5–15.4)	
**SEF: perceived difficulty of quitting (how hard to quit completely)^f^**						
Not at all or slightly	27.5 (23.8–31.6)	29.3 (22.1–37.8)	20.0 (19.0–21.4)	29.8 (26.4–33.3)	22.1 (21.0–23.3)	**<0.001**
Moderately	20.3 (17.0–24.1)	22.2 (17.3–28.0)	20.8 (19.6–22.1)	25.6 (22.1–29.3)	21.4 (20.3–22.5)	
Very or extremely	52.2 (47.8–56.5)	48.5 (40.4–56.5)	59.0 (57.6–60.5)	44.7 (40.9–48.6)	56.5 (55.1–57.8)	
**History of quitting**			**% (95% CI)**			
**Ever tried to quit**	70.3 (66.2–74.1)	59.8 (52.9–66.4)	64.0 (62.4–65.6)	54.1 (49.9–58.2)	63.1 (61.7–64.5)	**<0.001**
QA in the past 12 months*	42.1 (37.8–46.5)	23.8 (17.8–31.2)	27.7 (26.4–28.9)	24.3 (21.3–27.6)	28.2 (27.1–29.3)	**<0.001**
Cessation aids in last QA*	29.1 (24.6–34.0)	23.2 (16.1–32.4)	20.2 (18.8–21.6)	19.3 (16.0–23.2)	20.9 (19.7–22.2)	**0.002**
**Ever used e-cigarettes**	53.3 (49.0–57.6)	33.9 (26.6–42.0)	38.5 (37.0–39.9)	29.8 (26.4–33.4)	38.4 (37.1–39.6)	**<0.001**

a Missing from 1.1%;

b Missing from 6.7%;

c Missing among 7.2%;

d 7.9% of don’t know and 0.1% refused to answer were incorporated into ‘not planning to quit’;

e Missing from 4.1%;

f Missing from 1.8%;

HSI: Heaviness of Smoking Index; SEF: Self-efficacy; QA: quit attempt;

* Assessed among smokers who ever made a quit attempt, cessation aids included: behavioural support, use of websites, pharmacotherapy, and e-cigarettes.

### Dependence levels

The great majority of smokers smoked cigarettes daily (90.9%), and had moderate HSI levels (mean=2.4) (Table 1). A large minority (41.0%) considered themselves to be very addicted. In the unadjusted analyses, the flavour of the preferred cigarette brand was significantly associated with all measures of cigarette dependence (Tables 1 and 2, unadjusted Model 1). Menthol cigarette smokers had the lowest levels of daily smoking, a lower HSI index, were less likely to smoke within 30 min of waking, and considered themselves less addicted. Smokers of other flavoured cigarettes had a dependence profile more similar to that of unflavoured tobacco smokers. In the fully adjusted analysis (Table 2, Model 3) with unflavoured tobacco being the reference group, menthol, other flavoured, and no usual flavour cigarette users were significantly less likely to smoke daily (AOR=0.47, 95% CI: 0.32–0.71; 0.33, 0.15–0.77; 0.34, 0.22–0.51, respectively). Menthol and no usual flavour smokers were less likely to smoke within 30 min of waking (AOR=0.52, 95% CI: 0.43–0.64; 0.66, 0.55–0.80, respectively) or to consider themselves to be very addicted to cigarettes (AOR=0.74, 95% CI: 0.59–0.94; 0.65, 0.52–0.78, respectively).

**Table 2:** Flavour of cigarettes and smoking and cessation profile (Ref. ‘unflavoured tobacco’)

*Outcome of interest*	*Model 1 OR ( 95% CI)*	p*	*Model 2^a^ AOR ( 95% CI)*	p*	*Model 3^b^ AOR ( 95% CI)*	p*
**Cigarette dependence**						
**Smoking daily vs not^c^**						
Menthol	**0.24 (0.19–0.31)**	**<0.001**	**0.37 (0.25–0.54)**	**<0.001**	**0.47 (0.32–0.71)**	**<0.001**
Other flavoured	**0.53 (0.31–0.91)**		0.49 (0.23–1.00)		**0.33 (0.15–0.77)**	
No usual flavour	**0.25 (0.20–0.31)**		**0.41 (0.29–0.60)**		**0.34 (0.22–0.51)**	
**Cigarette dependence**						
**Smoke within 30 min of waking^d^**						
Menthol	**0.46 (0.38–0.56)**	**<0.001**	**0.54 (0.44–0.66)**	**<0.001**	**0.52 (0.43–0.64)**	**<0.001**
Other flavoured	**0.80 (0.63–1.23)**		**0.86 (0.62–1.21)**		**0.86 (0.59–1.24)**	
No usual flavour	**0.72 (0.60–0.86)**		**0.72 (0.59–0.86)**		**0.66 (0.55–0.80)**	
**HIS score** ≥**4**^**c**^						
Menthol	**0.47 (0.235–0.62)**	**<0.001**	**0.60 (0.45–0.89)**	**0.003**	**0.63 (0.47–0.83)**	0.011
Other flavoured	0.88 (0.60–1.28)		0.85 (0.60–1.22)		0.80 (0.54–1.19)	
No usual flavour	1.07 (0.88–1.31)		1.07 (0.87–1.30)		1.03 (0.84–1.26)	
**Consider themselves very addicted**						
Menthol	**0.51 (0.42–0.62)**	**<0.001**	0.80 (0.64–1.01)	**<0.001**	**0.74 (0.59–0.94)**	**<0.001**
Other flavoured	**0.67 (0.47–0.97)**		0.63 (0.43–1.02)		0.71 (0.47–1.08)	
No usual flavour	**0.56 (0.48–0.66)**		**0.63 (0.52–0.77)**		**0.65 (0.52–0.78)**	
**Quitting plans and self-efficacy**						
**Plan to quit in the next 6 months vs all other**						
Menthol	**1.82 (1.47–2.25)**	**<0.001**	**1.32 (1.05–1.68)**	**0.032**	1.05 (0.82–1.35)	0.978
Other flavoured	0.82 (0.52–1.26)		0.76 (0.50–1.16)		0.99 (0.65–1.50)	
No usual flavour	1.03 (0.85–1.25)		0.90 (0.73–1.12)		1.02 (0.82–1.28)	
**Very or extremely sure they could quit vs all other**						
Menthol	**1.74 (1.44–2.10)**	**<0.001**	1.20 (0.97–1.48)	**0.002**	1.11 (0.90–1.38)	**0.003**
Other flavoured	1.06 (0.77–1.45)		0.95 (0.69–1.30)		0.99 (0.72–1.36)	
No usual flavour	**1.60 (1.36–1.89)**		**1.40 (1.17–1.68)**		**1.40 (1.17–1.68)**	
**Expect quitting to be not at all or slightly difficult vs all other**						
Menthol	**1.50 (1.23–1.85)**	**<0.001**	1.03 (0.81–1.31)	**<0.001**	1.28 (1.00–1.65)	**<0.001**
Other flavoured	**1.65 (1.12–2.43)**		**1.87 (1.19–2.92)**		**1.82 (1.20–2.77)**	
No usual flavour	**1.68 (1.40–2.01)**		**1.48 (1.21–1.82)**		**1.46 (1.19–1.80)**	
**Prior cessation behaviour**						
**Ever made a QA**						
Menthol	**1.33 (1.09–1.62)**	**<0.001**	**1.16 (1.03–1.32)**	**<0.001**	1.13 (0.91–1.40)	**0.001**
Other flavoured	0.84 (0.63–1.12)		**1.21 (1.03–1.42)**		0.97 (0.69–1.36)	
No usual flavour	**0.66 (0.56–0.79)**		1.28 (0.78–2.09)		**0.69 (0.58–0.84)**	
**QA in the past 12 months**						
Menthol	**1.90 (1.57–2.29)**	**<0.001**	**1.45 (1.18–1.78)**	**<0.001**	1.20 (0.98–1.50)	0.045
Other flavoured	0.82 (0.56–1.19)		0.77 (0.54–1.09)		0.92 (0.64–1.32)	
No usual flavour	0.84 (0.70–1.01)		**0.73 (0.60–0.89)**		0.81 (0.66–1.00)	
**Last QA: used any cessation support^c^**						
Menthol	**1.62 (1.27–2.07)**	**0.001**	1.35 (1.03–1.77)	0.043	1.01 (0.75–1.36)	0.669
Other flavoured	1.20 (0.75–1.91)		1.08 (0.69–1.71)		1.35 (0.83–2.17)	
No usual flavour	0.95 (0.74–1.22)		0.81 (0.61–1.07)		1.06 (0.79–1.42)	
**Ever used e-cigarettes**						
Menthol	**1.83 (1.52–2.20)**	**<0.001**	**1.56 (1.28–1.92)**	**<0.001**	**1.26 (1.00–1.57)**	**<0.001**
Other flavoured	0.82 (0.58–1.17)		0.85 (0.58–1.24)		1.12 (0.73–1.74)	
No usual flavour	**0.68 (0.57–0.81)**		**0.57 (0.47–0.70)**		**0.66 (0.54–0.82)**	

AOR: adjusted odds ratio; HSI: Heaviness of Smoking Index; QA: quit attempt;

a Model 2: adjusted for sex, age, education, and HSI;

b Model 3: adjusted for sex, age, education, HSI, and country;

c Model was not adjusted for HSI;

d OR and AOR for a model with the variable dichotomised into ‘smokes within 5 min/all other’ were comparable, but not significant.

* p-values are for the overall model effect of the variable cigarette flavour in each model.

### Plans and self-efficacy to quit

A considerable proportion (39.6%) of smokers were not planning to quit, and only 21.0% planned to quit in the next 6 months. The majority had low quitting self-efficacy (61.4% were not at all or slightly sure they could succeed at quitting and 56.5% believed quitting would be very or extremely hard) (Table 1). In the bivariate analysis, cigarette flavour was significantly associated with plans and self-efficacy to quit, with unflavoured tobacco users having the lowest self-efficacy and menthol tobacco users having more immediate plans to quit (Table 1; Model 1 in Table 2). In the fully adjusted analysis (Table 2, Model 3), cigarette flavour was not significantly associated with plans to quit. Cigarette flavour remained significantly predictive of self-efficacy at quitting, with no usual flavour smokers having higher odds for higher self-efficacy for being sure they could quit (AOR=1.40, 95% CI: 1.17–1.68) or not considering quitting as difficult (AOR=1.46, 95% CI: 1.19–1.80). Other flavoured cigarette users had higher odds of expecting quitting not to be difficult (AOR=1.81, 95% CI: 1.20–2.77).

### Prior cessation behaviour

Over 63% of smokers had ever made a quit attempt, 28.2% had made a quit attempt in the past 12 months, and 20.9% had used some cessation aids (except e-cigarettes) during their last quit attempt, and 38.4% had ever tried e-cigarettes (Table 1). In the unadjusted and partially adjusted models (Table 1, Models 1 and 2 in Table 2), cigarette flavour was significantly associated with these prior cessation behaviours. These behaviours were significantly more prevalent among menthol smokers in comparison to unflavoured tobacco smokers. The relationships between cigarette flavour and cessation behaviours were attenuated once country was controlled in the fully adjusted models (Model 3, Table 2); no usual flavour smokers were less likely to have ever made a quit attempt (AOR=0.69, 95% CI: 0.58–0.84) or have ever used e-cigarette (AOR=0.66, 95% CI: 0.54–0.82). Menthol cigarette users were more like to have ever used e-cigarettes (AOR=1.26, 95% CI: 1.00–1.57).

## DISCUSSION

This cross-sectional multi-country study is among the first to explore flavoured cigarette smoking and cessation patterns, behaviours, quitting motivation and self-efficacy among European MFC smokers. The preference for flavoured cigarettes, and particularly menthol cigarettes, was significantly associated with lower smoking dependence, higher quitting self-efficacy, having made an attempt to quit in the past 12 months, having used any cessation aids, and ever use of e-cigarettes, after we accounted for sociodemographic and dependence characteristics. However, part of this association was accounted for by the country, since its inclusion in the model attenuated the association between cigarette flavours and cessation behaviours. Since many of the characteristics of menthol cigarette smokers are associated with quitting behaviours and success, these results suggest that the upcoming EU-wide TPD ban on additives may create an opportunity for increased cessation rates, but not all countries may see the same degree of benefit.

Smokers of different flavoured cigarettes were not a homogeneous group and were characterised by different levels of dependence levels, prior cessation behaviours as well as quitting self-efficacy. These differences are also consistent with findings from earlier analyses based on the same dataset, which demonstrated differences in the sociodemographic profile of smokers of menthol and other flavoured cigarettes, as well as their attitudes towards tobacco control measures^13^.

Importantly, compared to smokers of unflavoured tobacco, smokers of menthol cigarettes tended to be significantly less nicotine dependent, including as assessed with time to first cigarette (a good indicator of dependence for menthol cigarette smokers^20^) and viewed themselves as less heavily addicted. This is an important finding, as lower dependence is a consistent predictor of cessation success when quit attempts are made^48,49^. Interestingly, these findings are in contrast to many of the earlier observations on menthol smokers in the US suggesting similar^22^ or higher dependence levels^14,17^, which may also be contributing to greater difficulties with quitting among this group^19,50-52^. The difference in findings may be due to the fact that different brands of menthol cigarettes may contain different levels of nicotine, and the number of cigarettes smoked per day may not reflect the actual intensity of smoking and thus the actual nicotine intake^6,19^. Additionally, social idiosyncrasies might also play a role in the US, where high levels of menthol smoking are concentrated almost entirely among minority populations, and in particular among African-American smokers, who are nearly 11 times more likely to use menthol cigarettes than White smokers^53^.

In unadjusted analyses, cigarette flavour was significantly associated with plans to quit, self-efficacy, and prior cessation behaviour. Menthol cigarette smokers tended to be more likely to plan to quit in the next 6 months, had higher self-efficacy, and engaged with more cessation-related behaviours, all of which are associated with future cessation success^42,48,54^. They also had higher odds of ever use of e-cigarettes. These observations suggest that menthol smokers in Europe might be more likely to succeed if they attempt to quit. However, while a ban on menthol and other flavourings could trigger a quit attempt, it could also lead to alternative behaviours undesirable from a tobacco control point of view. In fact, MFC smokers have declared a gamut of intentions following the ban, ranging from quitting or reducing the amount smoked, to switching to another brand, or even finding a way to get the banned product – with the latter two options indicated most frequently^13^.

Furthermore, many of the differences in plans to quit, self-efficacy, and cessation behaviours were diminished in the adjusted analyses, especially after controlling for country. Important differences exist between European countries in implementation of tobacco control measures and offering cessation support^55,56^. These differences affect opportunities of smokers to engage with evidence-based support and are also likely to influence smokers’ cessation efforts, plans and self-efficacy^57,58^. Thus, while smokers of different flavoured cigarettes may differ on important characteristics related to smoking and cessation, the circumstances in their countries remain important predictors. Countries should implement best practice strategies in tobacco control and offer smoking cessation support.

The EU TPD ban on additives, including menthol and other flavourings, will have an impact on a significant minority of current smokers in Europe, of which menthol smokers are the largest group. The present findings show that smokers of menthol cigarettes demonstrate many characteristics that are positively associated with initiating quitting and remaining abstinent. Therefore, if menthol smokers are prompted to make a quit attempt, their success rates may be relatively higher^48,59^. Efforts at reducing smoking prevalence in countries such as England and Poland, where the proportion of MFC smokers is particularly high (almost 15%), as well as among female smokers, among whom MFC use (13.7%) is almost twice as high as among men, are particularly well placed to benefit from the ban^13^.

However, an earlier study assessing the plans of the same population of smokers in Europe in response to the TPD ban suggests that only a minority would consider quitting smoking due to the ban, and even more smokers are still not sure how they will react to the ban^13^. This study suggests that activities focused on supporting menthol smokers to initiate quitting during the implementation of the TPD ban on additives could increase cessation and lead to favourable public health outcomes. Such activities could be delivered by governmental and healthcare institutions, but also non-governmental organisations and charities. At the same time, the relationship between cessation behaviours and countries that emerged in this study provides an indication that the biggest challenges, but also opportunities for TPD ban on additives, will be in countries where smokers make fewer attempts, have lower motivation to quit, and use fewer cessation aids.

### Strengths and limitations

This was the first study to comprehensively characterise the smoking and quitting behaviours and perceptions of MFC smokers in European countries. We were also able to compare these smokers on a range of characteristics that were shown to be associated with cessation behaviour and success in previous studies. However, the study has some limitations. First, the study was cross-sectional and thus precludes any causal interpretation of associations found. Future research will need to assess cessation behaviour and outcomes prospectively. Second, all respondents were current smokers, which precluded making comparisons between successful and unsuccessful quit attempts and assessing predictors of cessation outcomes. Third, the wording of survey questions did not allow for making a distinction between smokers who used menthol or other flavoured cigarettes predominantly or only in conjunction with unflavoured tobacco. Fourth, residual confounding due to unassessed or imperfectly measured confounders is possible, such as the duration of menthol cigarette use among the sample, which was shown previously to be associated with smoking and quitting behaviour^26^. Finally, there exist important differences in prevalence of use of different flavours of cigarettes^13^ and in cessation behaviour between European countries^57^, and interaction between variables may exist that pose challenges to interpreting the main effects. However, due to too few cases of menthol and flavoured cigarette users in each country, assessing such interactions is not possible. Future research should explore the differences between different tobacco users across countries.

## CONCLUSIONS

In the eight European countries there seem to be important differences in the smoking and cessation profile, behaviours, and self-efficacy between smokers of menthol, flavoured, unflavoured tobacco, and those with no usual brand. The lower dependence levels among menthol and flavoured cigarette users is particularly encouraging as lower dependence is predictive of cessation success. In the light of the EU TPD ban on characterising flavours in tobacco products in the EU, tobacco control activities should focus on increasing quit attempts of menthol smokers, which could translate into greater cessation rates.
